# The Education of Nervous Children

**Published:** 1895-04-27

**Authors:** 


### The Education of Nervous Children.

The Lumleian Lectures recently delivered by Dr. Blandford on "The Diagnosis, Prognosis, and Prophylaxis of Insanity" touch upon topics of far wider than merely professional interest, and incidentally tend to show how all-embracing is the science of medicine.

Dr. Blandford insists upon the great and preponderating influence of heredity in the production of insanity. He also points out that the true prophylaxis of insanity is to be found in the proper regulation of marriage. Many, he says, have hoped that legislation may some day concern itself with this subject and interfere to prevent marriages between people with neurotic tendencies. How chimerical such hopes are, however, we may judge from the fact that so far legislation will not even annul the bond which ties a man or woman to a partner, perhaps quite young, who is hopelessly insane and may remain so for the remainder of a long life. It is necessary then to turn to the practical, and consider what may best be done to conserve the mental health of those who are born with a tendency to mental deficiency or disease.

In early days food and plenty of it, sleep and plenty of that also, and careful guarding from all causes of nerve disturbance are to be ensured. It is difficult to exaggerate the evil that may be worked upon a nervous child by the tales of ghosts, robbers, bogies, or policemen, with which many children are continuously indulged. The inculcation of the precepts of religion also too often becomes the means by which terrifying ideas are implanted in the infant mind, much to the disturbance of that peacefulness during the hours of rest which is essential to its normal development. It is pitiful to think of the number of children who instead of dropping into dreamless slumber look forward to bedtime as a time of terror, bury their faces in the bedclothes, and only when exhausted fall asleep haunted by visions of a constant all-seeing eye, by dread of unending torments for each unforgiven, maybe forgotten, little fault, and by fear of lightning and other natural phenomena which they have been taught are instruments of wrath. From all such thoughts the developing brain should be protected. But later on, when education, as it is now understood, begins, the child among whose ancestors neuroses have prevailed requires even more protection, for every influence is often brought to bear to disturb his mental equilibrium. If he is dull and backward he is punished, if he is bright, intelligent, and precocious he becomes the pride of his teachers and is set to win scholarships for the glory of his school. In either case he is exposed to the very turmoil of mind from which he should be most protected. In the choice of a profession again the young man is beset almost everywhere by the terrors and evils of competitive examinations; and this brings us to one of the most important points in the prevention of insanity in those predisposed to it, viz., the fitting of the work of life to the capacity of the individual. The ambitions of the parent, combined with the ill-regulated hopes, desires, and fancies of the son, often drive the latter to undertake responsibilities for which he is by no means fitted, and thus cause nervous break down which might well have been avoided. As Dr. Blandford says, "It is not difficult to point out the occupations which are unsuited to such a man; it is more difficult to say what is the most suitable. That will be best which entails least worry and responsibility, which does not involve money losses or great and sudden disappointments." But what sort of a life is this; where is it to be found; and who is to impose it on young men who are often of exceptional brilliancy and precocity? So far as the dullards are concerned, parents will be well advised not to push them, to accept the fact that they are mentally ill-developed, and to avoid placing them in positions involving responsibility and care. This is a blow to the father's pride, and that is all; the son will accept his lot. But the brilliant scion of a neurotic or of an insane family is not so easily dealt with, for at twenty-one he is his own master. All the more necessary is it then that care should be taken in the early training of those whose family history shows any taint of insanity. It is in infancy and childhood that most can be done, for when the time of manhood is arrived at a man is free, whatever his family history, and thus that careful regulation of the mode of life upon which the prophylaxis of insanity depends falls upon the very man who has received an heritage of instability of mind, and who may be difficult to control or to persuade. The outlook is not encouraging.

